# Security of quantum digital signatures for classical messages

**DOI:** 10.1038/srep09231

**Published:** 2015-03-18

**Authors:** Tian-Yin Wang, Xiao-Qiu Cai, Yan-Li Ren, Rui-Ling Zhang

**Affiliations:** 1 School of Mathematical Science, Luoyang Normal University, Luoyang. 471022, China; 2 Start Travel Collaborative Innovation center of Zhongyuan Economic area, Luoyang Normal University, Luoyang 471022, China; 3 School of Communication and Information Engineering, Shanghai University, Shanghai. 200444, China; 4 School of Information Technology, Luoyang Normal University, Luoyang 471022, China

## Abstract

Quantum digital signatures can be used to authenticate classical messages in an information-theoretically secure way. Previously, a novel quantum digital signature for classical messages has been proposed and gave an experimental demonstration of distributing quantum digital signatures from one sender to two receivers. Some improvement versions were subsequently presented, which made it more feasible with present technology. These proposals for quantum digital signatures are basic building blocks which only deal with the problem of sending single bit messages while no-forging and non-repudiation are guaranteed. For a multi-bit message, it is only mentioned that the basic building blocks must be iterated, but the iteration of the basic building block still does not suffice to define the entire protocol. In this paper, we show that it is necessary to define the entire protocol because some attacks will arise if these building blocks are used in a naive way of iteration. Therefore, we give a way of defining an entire protocol to deal with the problem of sending multi-bit messages based on the basic building blocks and analyse its security.

Digital signature (DS) is a fundamental cryptographic primitive, which has been frequently used in e-commerce and e-government to ensure both the integrity and the origin of a message. However, the degree of security provided by current classical digital signature (CDS) schemes generally depends on certain unproven assumptions related to the intractability of certain difficult mathematical problems, such as big number factorization problem^1^ and discrete logarithmic problem^2^. With the rapid development of quantum computing^3^, the security of such CDS schemes is seriously challenged.

Fortunately, quantum digital signature (QDS) provides a way of authenticating classical messages with information-theoretic security against forging and repudiation. Gottesman and Chuang introduced the concept of QDS in 2001, and proposed the first QDS scheme for classical messages based on quantum one-way functions^4^.

Recently, a novel QDS proposal for classical messages was put forth (named C-proposal hereafter), which has been implemented using phase-encoded coherent states of light in experiments^5^. However, it needs quantum memory like previous proposals, which makes it also unfeasible in practice with current technology. To deal with this problem, Dunjko et al gave the first practical QDS proposal for classical messages, in which quantum memory is no longer required^6^; in addition, this proposal has been implemented using just standard linear optical components and photodetectors^7^. Furthermore, Dunjko et al presented another two different QDS protocols for classical messages, which essentially only use the same experimental requirements as quantum key distribution^8^. Most important of all, in contrast with other DS schemes, this kind of proposals^5^,^6^,^7^,^8^ have an important advantage: the trusted authorities are not needed any longer.

These QDS proposals^5^,^6^,^7^,^8^ are basic building blocks, which only deal with the problem of sending single bit messages while no-forging and non-repudiation are guaranteed. For a long multi-bit message, it is only mentioned that the basic building blocks must be iterated, but the iteration of the basic building blocks still does not suffice to define the entire protocol, and therefore there still must be an additional set of rules which stipulate how disputes are resolved, or how validity of a long message is proven and so on.

In this paper, we show that it is necessary to define the entire protocol because some attacks will arise if these basic building blocks are used just in a naive way of iteration. Furthermore, based on the basic building blocks in these proposals^5^,^6^,^7^,^8^, we propose an entire protocol to deal with the problem of sending multi-bit messages, in which the rules on how to resolve disputes, and how to prove the validity of a multi-bit message and so on are given.

## Results

As mentioned above, these QDS proposals^5^,^6^,^7^,^8^ are basic building blocks, which only deal with the problem of sending single bit messages while no-forging and non-repudiation are guaranteed. For a long multi-bit message, it is only mentioned that the basic building block must be iterated, but the iteration of the basic building block still does not suffice to define the entire protocol. Specifically, some attacks will arise if these building blocks are used to deal with the problem of sending a multi-bit message in a naive way of iteration. Without loss of generality, we take three players' case of C-proposal as an example.

### The C-proposal

**Figure 1:**
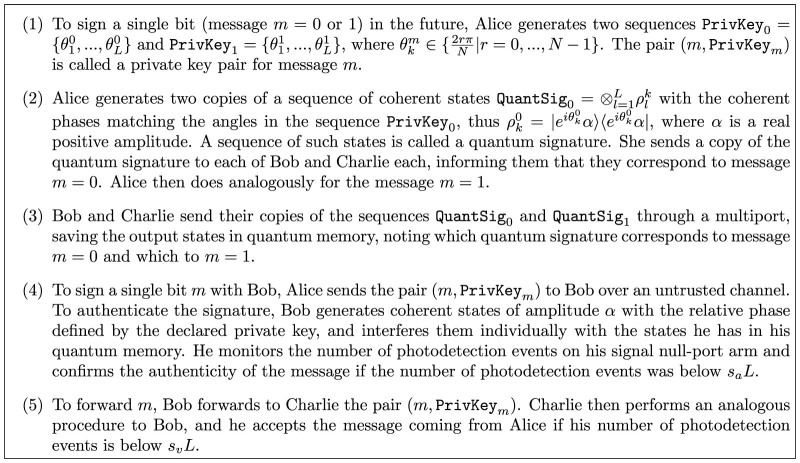
C-proposal. (1) To sign a single bit (message *m* = 0 or 1) in the future, Alice generates two sequences 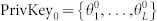 and 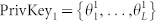, where 
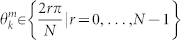
. The pair (*m*, PrivKey*_m_*) is called a private key pair for message *m*. (2) Alice generates two copies of a sequence of coherent states 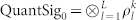 with the coherent phases matching the angles in the sequence PrivKey_0_, thus 
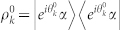
, where *α* is a real positive amplitude. A sequence of such states is called a quantum signature. She sends a copy of the quantum signature to each of Bob and Charlie each, informing them that they correspond to message *m* = 0. Alice then does analogously for the message *m* = 1. (3) Bob and Charlie send their copies of the sequences QuantSig_0_ and QuantSig_1_ through a multiport, saving the output states in quantum memory, noting which quantum signature corresponds to message *m* = 0 and which to *m* = 1. (4) To sign a single bit *m* with Bob, Alice sends the pair (*m*, PrivKey*_m_*) to Bob over an untrusted channel. To authenticate the signature, Bob generates coherent states of amplitude *α* with the relative phase defined by the declared private key, and interferes them individually with the states he has in his quantum memory. He monitors the number of photodetection events on his signal null-port arm and confirms the authenticity of the message if the number of photodetection events was below *s_a_L*. (5) To forward *m*, Bob forwards to Charlie the pair (*m*, PrivKey*_m_*). Charlie then performs an analogous procedure to Bob, and he accepts the message coming from Alice if his number of photodetection events is below *s_v_L*.

Before presenting the attacks, let us give a simple introduction of C-proposal, which can be described in Figure 1.

### The analysis of C-proposal

From C-proposal, it can be seen that if its basic building blocks are used to deal with the problem of sending a multi-bit message just in a naive way of iteration, and a signed multi-bit message (*M*, PrivKey*_M_*) (we will call it a message-signature pair hereafter) will be verified in the way of bit by bit, and there is no correlation among quantum signatures on signed message bits except that their labels are pre-determined and sequential. Furthermore, as mentioned in Ref. 8, a QDS protocol has two stages: a preparation stage (distribution) and a message stage. The distribution stage serves to establish the required classical-quantum (or fully classical) correlations, which can later, in the message stage, be used by the sender to transmit messages to the recipients. Additionally, no further communication with any of the other players is required when the sender (say Alice) sends a message-signature pair to a recipient, and both the transferal and the verification of the message-signature pair should no longer require any feedback from Alice at all; in addition, Alice may send a lot of different message-signature pairs to the recipient and other ones later (in the message stage). Therefore, the verifier Charlie knows neither the length of a signed message nor the initial label of quantum signature for the message sent by the recipient. These will give a chance for a dishonest recipient (say Bob) to forge an integrated message-signature pair by the following known-message attacks.

**Forgery attack 1.** Suppose that Bob has obtained a valid message-signature pair (*M*, PrivKey*_M_*) from Alice, where 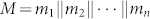, and 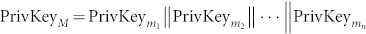, here || denotes the concatenation of bits or bit strings. He chooses some continuous bits from *M* (e.g., the first half bits) and the corresponding private keys from PrivKey*_M_*, which are denoted as (*M*′, PrivKey*_M_*_′_), where
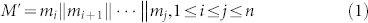
and
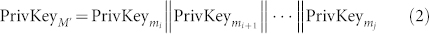
Then he sends the new message-signature pair (*M*′, PrivKey*_M_*_′_) to Charlie. It can be seen that the forged message-signature pair (*M*′, PrivKey*_M_*_′_) is a subset of the valid message-signature pair (*M*, PrivKey*_M_*) and each signed bit *m_k_* is not changed, *i* ≤ *k* ≤ *j*, i.e., *M*′ ⊆ *M*, PrivKey*_M_*_′_ ⊆ PrivKey*_M_*. Therefore, each bit-signature pair (*m_k_*, 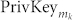) of (*M*′, PrivKey*_M_*_′_) matches the corresponding quantum signature 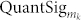 stored by Charlie, which means Bob's forgery introduces no error and therefore the forged message-signature pair (*M*′, PrivKey*_M_*_′_) will be accepted by Charlie. For example, suppose that Bob has received a message-signature pair (*M*, PrivKey*_M_*) from Alice, where *M* = “don't pay Bob 100$.” then Bob will be able to send Charlie the message *M*′ (*M*′ = “pay Bob 100$.”) and the corresponding PrivKey*_M_*_′_ to Charlie, claiming that it comes from Alice, where the initial “Don't” is omitted. For *M*′ ⊆ *M*, PrivKey*_M_*_′_ ⊆ PrivKey*_M_*, Charlie will accept that it comes from Alice and give 100$ to Bob.

**Forgery attack 2.** Suppose that Bob has obtained two valid message-signature pairs (*M*_1_, 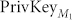) and (*M*_2_, 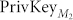) from Alice, where 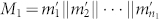, 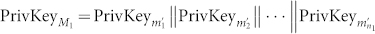, 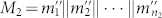, and 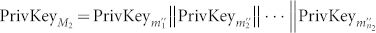. He chooses some continuous bits from *M*_1_ and *M*_2_ (e.g., the last half bits of *M*_1_ and the first half bits of *M*_2_) with their corresponding private keys to form a new message-signature pair (*M*″, PrivKey*_M_*_″_), where
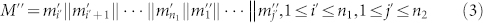
and
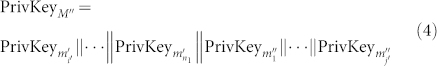
Then he sends the forged message-signature pair (*M*″, PrivKey*_M_*_″_) to Charlie. Clearly, 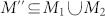, 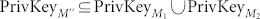, and therefore by similar analysis as that in forgery attack 1, the forged message-signature pair (*M*″, PrivKey*_M_*_″_) will also pass Charlie's verification.

It is noted that the label of quantum signature for the last bit of *M*_1_ and the label of quantum signature for the first bit of *M*_2_ must be successive in forgery attack 2, i.e., if the label of quantum signature 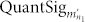 for 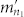 is *l*, then that for 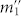 must be *l* + 1, which ensures the labels of quantum signature for the forged message-signature pair (*M*″, PrivKey*_M_*_″_) are sequential and Bob's deception is not detected by Charlie. Additionally, an outside adversary Eve also can forge a valid message-signature pair when the message-signature pairs are transmitted over an insecure channel. For example, she intercepts them when Alice sends message-signature pairs to a legal recipient, and then she forges a new message-signature pair by the way that Bob does in the above forgery attacks.

As mentioned in Refs. 9, 10, a signature scheme is broken if an opponent can do any of the following with a nonnegligible probability:

Universal forgery (total break), in which he/she can forge a signature for any message.

Selective forgery, in which he/she can forge a signature for a particular message chosen by him/her.

Existential forgery, where he/she can forge a signature for at least one message, but he/she has no control over the message whose signature he obtains, i.e., the message may be random or nonsensical.

However, if the basic building blocks in these proposals^5^,^6^,^7^,^8^ are used to deal with the problem of sending a multi-bit message in a naive way of iteration, a dishonest recipient or an outside adversary can successfully forge a valid signature for a particular message (chosen from a valid signed message by himself in advance) by the above known-message attacks. Furthermore, the forged message is not random or nonsensical in many cases. For example, if the signed message sent by Alice is a contract, forgery attack 1 allows Bob to delete some items that may be not beneficial to him, and forgery attack 2 allows him to add some new items from another one. Moreover, as a legal replacement for handwritten signatures, DS is not only used to send a message; in addition, the signatory of a signature scheme would like to feel that he/she may sign arbitrary documents prepared by others without fear of compromising his/her security, such as the case of a notary public who must sign more-or-less arbitrary documents on demand^10^. Therefore, it is a natural and reasonable assumption that an opponent may gain access to valid signatures for any messages of his/her choice (where each message may be chosen in a way that depends on the signatures of previously chosen messages), i.e., we should allow an opponent can do a forgery in the model of adaptive chosen-message attacks; in this case, the opponent can forge a valid signature on any message chosen by himself/herself in advance.

## Discussion

It has been shown that the iteration of the basic building blocks of dealing with the problem of sending single bit messages still does not suffice to define the entire protocol. Therefore, it is a necessary and significative work to study the problem of sending multi-bit messages based on the basic building blocks.

As we know, the main tasks of DS are to prevent impersonation, repudiation and message tampering in data transfer, of which the key is to guarantee the integrity of signed messages, i.e., any alteration of a signed message will be detected in the process of verifying. In these proposals^5^,^6^,^7^,^8^, nobody can forge a valid signature for a single message bit except with a negligible probability. Furthermore, the label of quantum signature for each message bit is predetermined and sequential. Therefore, if the start and the end of a signed message are tagged, i.e., both the initial label and the last one of quantum signatures for the signed message cannot be changed, whereby the integrity of a signed message can be guaranteed. To this goal, one way is that Charlie can acquire the two labels from the signatory Alice before verifying, but it needs some communications or feedbacks between them, which is obviously contradictory to the natural requirement for DS transferal and verification, and another way is that both the start and the end of a signed message are different from the message bits, meanwhile the signatures for them are not to be forged, which can be realized by a special encoding way. In the following, we propose an entire protocol to deal with the problem of sending multi-bit messages, in which the validity of the special encoding way to guarantee the integrity of a signed multi-bit message is proven.

## Methods

A method to define an entire protocol for dealing with the problem of sending a multi-bit message is described as follows.

- The preparation (distribution) stage is the same as that in these proposals^5^,^6^,^7^,^8^.
- In the message stage, Alice encodes each bit 0(1) of the message *M* by the codeword 000(010) before signing, i.e., 0 → 000, 1 → 010. Then she adds a special codeword 111 to both the start and the end of the message, in this way, the message *M* is encoded to 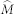. After that, Alice signs each bit 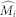 of 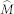 by using the signing block in these proposals^5^,^6^,^7^,^8^, where 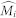 is the *i*th bit of 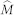. Finally, she sends the resulting message-signature pair (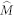, 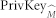) to Bob, where 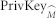 is the concatenation of the signature 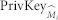 for the bit 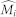 of 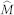. If each signature 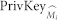 for the bit 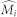 of 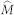 passes his verification, Bob confirms the authenticity of the message 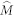.
- In the verifying stage, when receiving the resulting message-signature pair (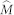, 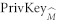) forwarded by Bob, Charlie firstly checks whether all codewords are legal, i.e., all codewords in the message bit sequence 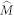 should be 000 or 010 except that the start and the end are the codeword 111. If it is not so, Charlie thinks the message *M* has been tampered with and rejects it. Otherwise, he continues to verify the validity of the signed message *M* by the same way as that in the corresponding proposal^5^,^6^,^7^,^8^. If each bit 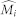 of the signed message 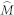 passes his verification, Charlie decodes 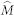 to *M* and accepts it coming from Alice and has not been tampered with; otherwise, he rejects it.

By this way, if Bob wants to forge a new valid message-signature pair by forgery attack 1 or forgery attack 2, he must find at least one new codeword 111 in the encoding bit sequence 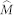 while guaranteeing there is no illegal codeword in the forged message. Nevertheless, it is not possible. To draw this conclusion, some necessary lemmas should be proven.

**Lemma 1** Suppose that 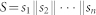, *s_i_* ∈ {000, 010}, *i* = 1, 2, …, *n*, is a bit sequence, then 111 ∉ *S*.

The conclusion of this lemma is obvious, but it implies that if each bit 0(1) of a message *M* is encoded by the codeword 000(010), then it is impossible to appear a codeword 111 in the corresponding encoding sequence. For example, let a message *M* = 01011001, then the message *M* is encoded to the bit sequence *S* = 000010000010010000000010, we cannot find a codeword 111 in the bit sequence *S*.

**Lemma 2** Suppose that 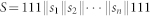, *s_i_* ∈ {000, 010}, *i* = 1, 2, …, *n*, is a bit sequence, it is impossible to find a sequence 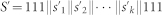, 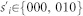, *i* = 1, 2, …, *k* such that *S*′ ⊆ *S* except *S*′ = *S*.

Proof. By Lemma 1, 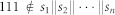. In addition, both the first bit and the last one of the codewords 000 and 010 are 0, thus it is impossible to find a new codeword 111 by the way of taking one bit from a message codeword 000 or 010 and two bits from the codeword 111, or taking two bits from a message codeword 000 or 010 and one bit from the codeword 111. Therefore, it is impossible to find a new codeword 111 in the middle of the bit sequence *S*, and hence we cannot find a sequence 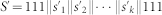, 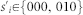, *i* = 1, 2, …, *k* such that *S*′ ⊆ *S* except *S*′ = *S*. For example, let *S* = 111000010010000010111, obviously, there is no codeword 111 in the middle of the bit sequence *S*, and therefore it is impossible to find a sequence 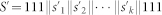, 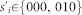, *i* = 1, 2, …, *k* such that *S*′ ⊆ *S* except *S*′ = 111000010010000010111.

**Lemma 3** Suppose that 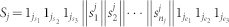, 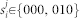, *i* = 1, 2, …, *n_j_*, *j* = 1, 2, …, *l*, it is impossible to find a sequence 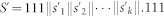, 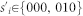, *i* = 1, 2, …, *k* such that 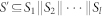 except *S*′ = *S_j_*, *j* = 1, 2, …, *l*.

Proof. When *l* = 1, this lemma reduces to Lemma 2.

When *l* = 2,
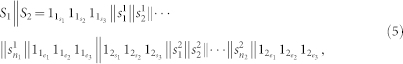
in this case, in order to find a sequence 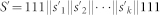, 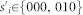, *i* = 1, 2, …, *k* such that 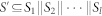, it is necessary to find at least one new codeword 111. By lemma 2 and Formula (5), the new codeword 111 can be only chosen from 
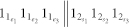
; if we choose 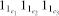, we must choose 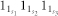 as the start codeword, but in the case, *S*′ = *S*_1_; otherwise, there must exist at least one codeword 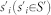 such that 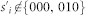. If we choose 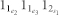, whether we choose 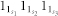 or 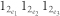 as another codeword 111, obviously, there must exist at least one codeword 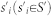 such that 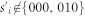; if we choose 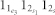 or 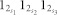, we will face the same difficult. Therefore, in any case, it is impossible to find a sequence 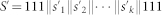, 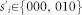, *i* = 1, 2, …, *k* such that *S*′ ⊆ *S*_1_||*S*_2_ except *S*′ = *S_j_*, *j* = 1, 2.

Suppose that when *n* = *l* − 1, this conclusion is right. When *n* = *l*, let 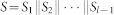, according to the former assumption, it is impossible to find a sequence 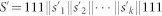, 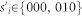, *i* = 1, 2, …, *k* such that *S*′ ⊆ *S* except *S*′ = *S_j_*, *j* = 1, 2, …, *l* − 1. By similar analysis as *l* = 2, we can get that it is impossible to find a sequence 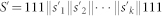, 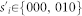, *i* = 1, 2, …, *k* such that *S*′ ⊆ *S*||*S_l_* except *S*y = *S_j_*, *j* = 1, 2, …, *l*.

As a result, it is impossible to find a sequence 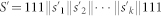, 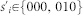, *i* = 1, 2, …, *k* such that 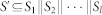 except *S*′ = *S_j_*, *j* = 1, 2, …, *l*. We also can give an example to show that. Let *S*_1_ = 111000000111, *S*_2_ = 111000010111, *S*_3_ = 111010010111, then *S*_1_||*S*_2_||*S*_3_ = 111000000111111000010111 111010010111, from the bit sequence 111000000111111000010111111010010111, it can be seen that we cannot find a sequence 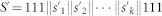, 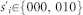, *i* = 1, 2, …, *k* such that *S*′ ⊆ *S*_1_||*S*_2_||*S*_3_ except *S*′ = 111000000111, 111000010111 or 111010010111.

From Lemmas 1, 2 and 3, we can conclude that even if an opponent has obtained a lot of message-signature pairs, he/she cannot forge a new valid message-signature pair by forgery attack 1 or forgery attack 2. It is noted that if the opponent can forge a bit-signature pair, he/she can forge a valid message-signature pair by forgery attack 1 or forgery attack 2 because he/she can forge a new codeword 111. Nevertheless, in C-proposal, it has been proven that the probability of forging a bit-signature pair is
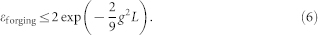
By simple computation, we can get that the probability of forging a signature for a new codeword 111 is not more than *ε*_forging_. Furthermore, the probability *ε*_forging_ is exponentially close to 0 with the increase of the parameter *L*. Consequently, if a large parameter *L* is chosen, the probability of forging a signature for a new codeword 111 is negligible.

Therefore, if the basic building blocks of dealing with the problem of sending single bit messages is secure against forging, our method can effectively guarantee the integrity of signed messages in the sense that it can prevent currently known attacks.

Finally, it should be noted that our method does not influence the security of QDS against repudiation, but it may be only one of several possibilities to guarantee the integrity of signed messages.

## Author Contributions

T.Y. and R.L. analysed the security of C-proposal and proposed the forgery attacks. T.Y., X.Q. and Y.L. proposed the way to guarantee the integrity of signed messages. T.Y. and X.Q. wrote the main manuscript text. All authors reviewed the manuscript.
